# Supplementary material for: 7T HR FID-MRSI Compared to Amino Acid PET: Glutamine and Glycine as Promising Biomarkers in Brain Tumors
Source: Cancers (Basel). 2022 Apr 26;14(9):2163. doi: 10.3390/cancers14092163 (PMC9101868; doi:10.3390/cancers14092163)
Supplement: Supplementary file 1 [file cancers-14-02163-s001.zip › cancers-1661986-supplementary.pdf]

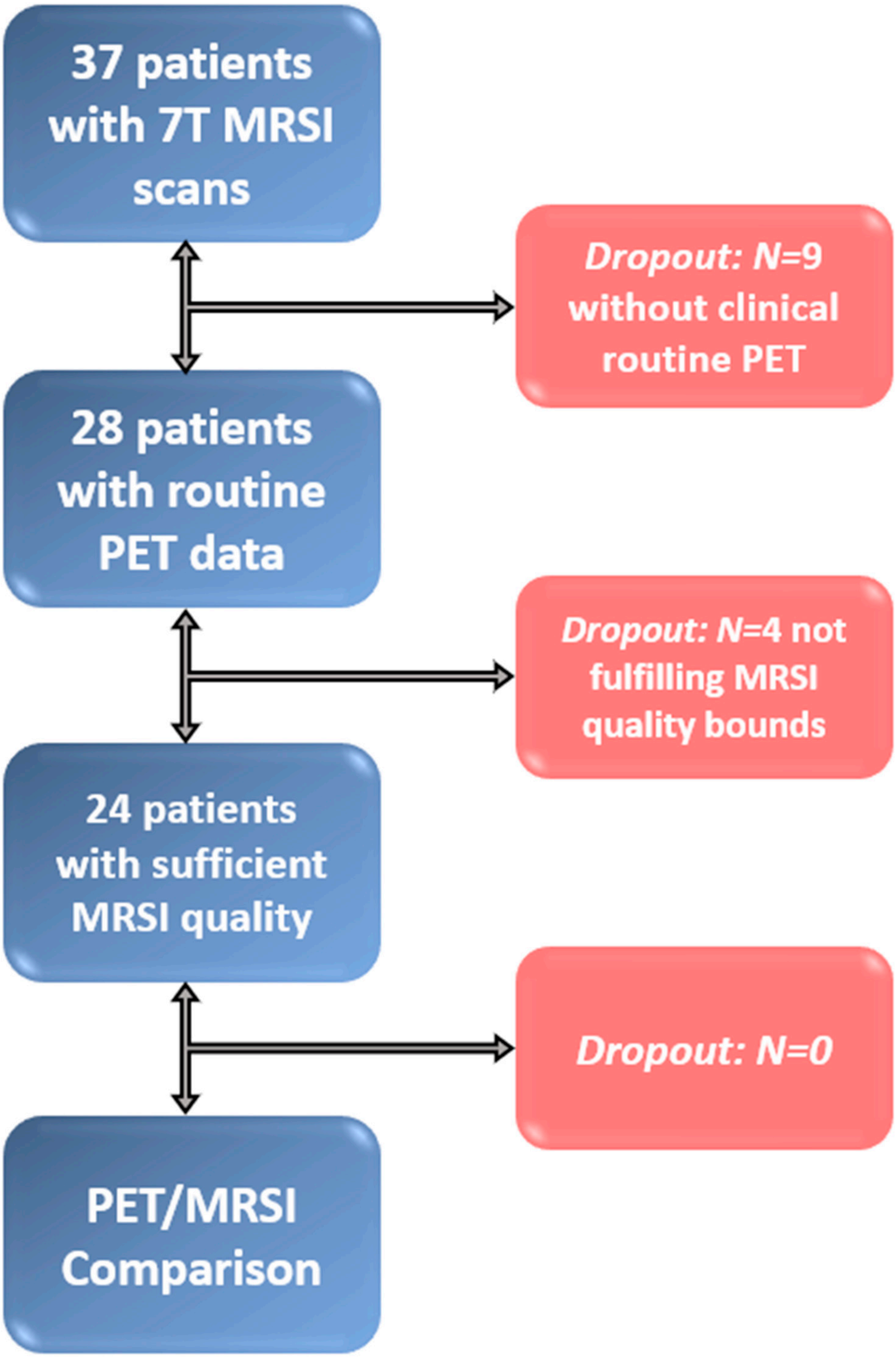

Figure S1. Patient recruitment.

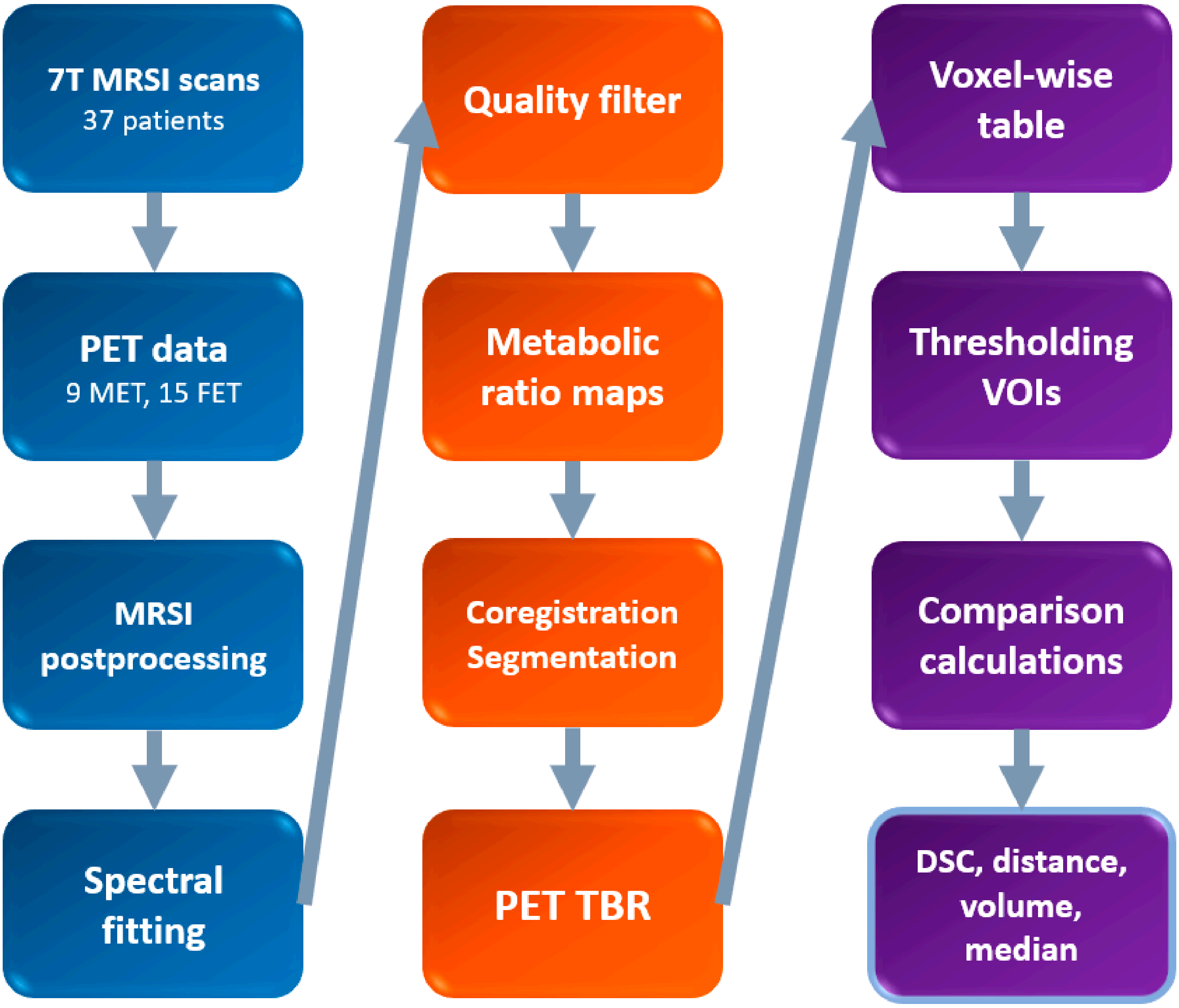

Figure S2. Workflow.

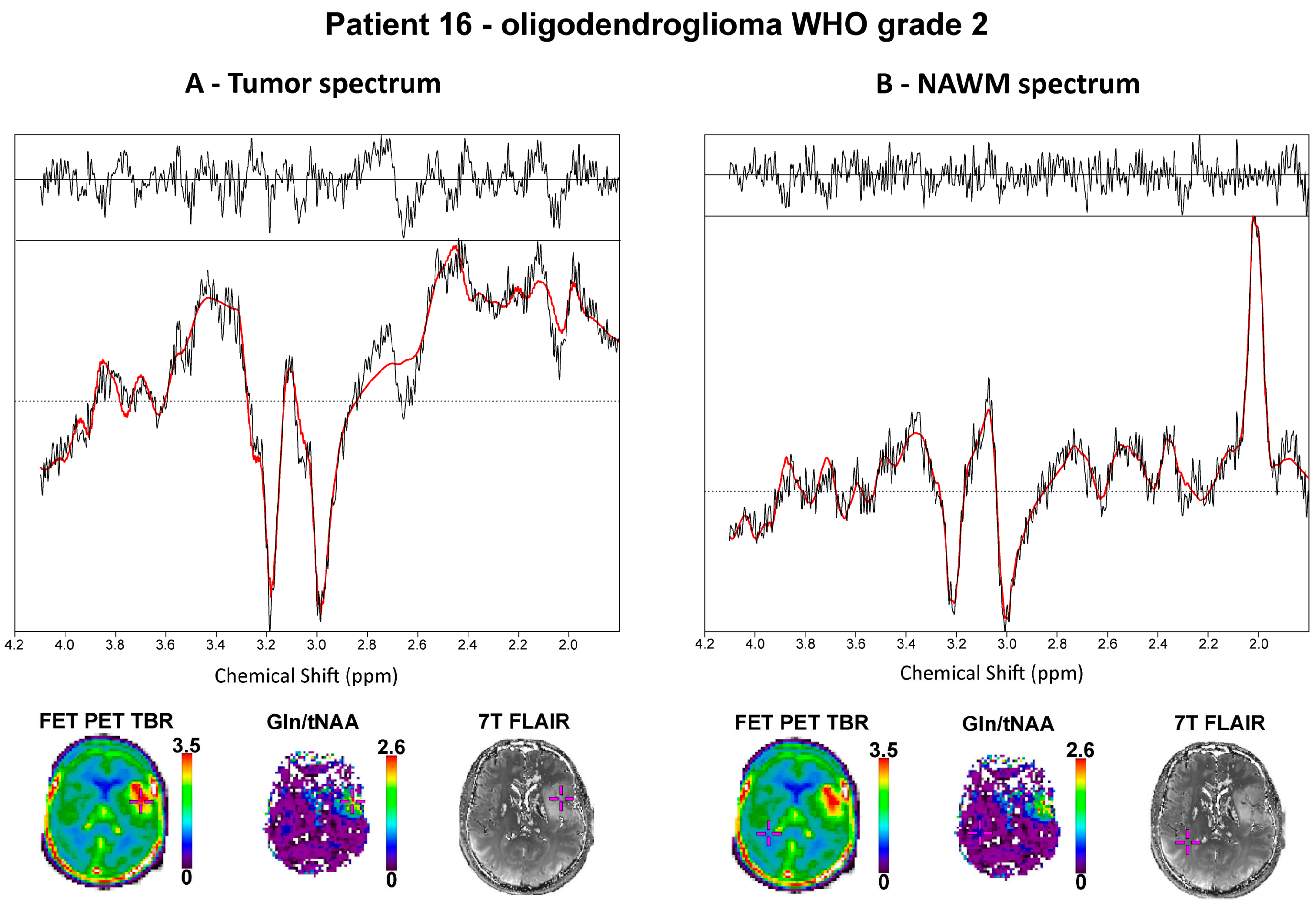

Figure S3. Example spectra.
